# Childbirth fear, birth-related mindset and knowledge in non-pregnant women without birth experience

**DOI:** 10.1186/s12884-022-04582-6

**Published:** 2022-03-24

**Authors:** Lisa Rublein, Beate Muschalla

**Affiliations:** Technische Universität Braunschweig, Psychotherapy and Diagnostics, Institute of Psychology, Humboldtstraße 33, 38106 Braunschweig, Germany

**Keywords:** Birth, Childbirth fear, Medical birth mindset, Natural birth mindset, Knowledge, Nulliparous, FOC, Fear of childbirth

## Abstract

**Background:**

Childbirth fear and interventions during childbirth might be related to the mindset and knowledge non-pregnant women have regarding childbirth. Non-pregnant women before their first birth experience may be particularly at risk for childbirth fear.

**Methods:**

The present study examined the expressions and associations of birth-related mindset, knowledge, and fear among 316 young, non-pregnant women without birth experience. They participated in a cross-sectional online study and completed the Childbirth Fear Prior to Pregnancy, the Mindset and Birth Questionnaire, and a birth knowledge test.

**Results:**

Most women (44%) had a natural mindset and low fear, 29% had a medical mindset and low fear, 8% natural mindset and higher fear, and 19% medical mindset and higher fear.

There were no differences in knowledge between the four groups. Some gaps in knowledge appeared concerning signs of beginning birth, and non-medical approaches to pain relief. From women with natural mindset and low childbirth fear, a higher percentage (13%) has already watched a birth, as compared to the other groups.

Natural mindset was associated with lower childbirth fear, whereas knowledge was independent from childbirth fear. Higher knowledge was low associated with natural mindset. Mindset and childbirth fear were independent from age and education degree.

**Conclusions:**

Gynecologists, midwifes and other health professionals may develop an awareness for birth as a natural event in their non-pregnant patients, and take birth-related fear into account in their counseling, with focus on women’s self-efficacy and non-medical approaches to pain relief.

## Childbirth fear, natural or medical mindset, birth-related knowledge

Along with scientific and medical advance during the past decades, childbirth has on the one hand become safer than ever before in many (but not all) industrialized countries. But, on the other hand, it is still coming along with many uncertainties. The natural risks of birth may come along with (in most cases normal) worries in 80% of pregnant women. But in some cases, there can be stronger childbirth-related fears with the tendency to seek for risk reduction, e.g. by cesarian section [1].

Until now evidence on childbirth fear and related characteristics is available for pregnant women. However, childbirth fear may also occur in women before pregnancy. Evidence on this group is rare. In this present study, we investigate childbirth fear as a continuous phenomenon, as self-rated anxiety on different aspects of birth, in non-pregnant women. Accordingly, research on primary childbirth fear, i.e. childbirth fear in non-pregnant women without birth experience of a viable child (analogous to primary tokophobia [2]), will add to the evidence. This present study aims to add data on relationships of primary childbirth fear, birth-related natural or medical mind set and birth-related knowledge. To have an idea about how many young women are affected from childbirth fear, and which accompanying characteristics in terms of birth-related mind set and knowledge they have, will be helpful for professionals who give counseling to young women before pregnancy.

### Childbirth fear

How many women are affected from childbirth fears? Childbirth fear is a dimensional phenomenon and can appear in different phenotypes. Research found rates of slight worries in 80% of pregnant women [1]. 20% of pregnant women perceive more specific worries with higher intensity. About 10% of nulliparous or parous women suffered from severe childbirth fear [3]. In a European cohort study from six countries, 5079 (78.7%) pregnant women were without childbirth fear, and 713 (12.3%) suffered from childbirth fear [4]. Specific, clinically relevant birth-related phobias which were assessed with structured diagnostic interviews, are more seldom: they have been found in 1.5% of pregnant women [5]. Tokophobia (phobic avoidance of the delivery) was most seldom with 0.03% [5].

From non-pregnant women, rates of clinically relevant childbirth fear have been reported for 25.9% [6]. Rates of childbirth fear in the different studies may vary due to different diagnostic instruments and cut offs. In conclusion one can expect that about 10–20% of pregnant or non-pregnant women before birth suffer from childbirth fears which need further clinical investigation.

Childbirth fear in non-pregnant women was found to be accompanied by postponing pregnancy planning, high expectation of labor pain, high trait anxiety, high psychical anxiety symptoms [6]. Young women studying health sciences were less affected from childbirth fear as compared to their female colleagues from social sciences and humanities. The investigated non-pregnant women reported that they had received most information about childbirth from their family and least often from professional books [6].

### Medicalization of obstetrics and natural and medical mindset

Along with increased safety of birth, a widespread medicalization of obstetrics was to be observed in the past decades. While births historically have long occurred out-of-hospital and without medical supervision, they have now become a medical event: Births usually take place in hospitals with the use of various medical interventions [7, 8]. This brings on the one hand the advantage of being able to deal effectively with complications and reduce perinatal mortality and morbidity [9]. On the other hand, the extensive medicalization of obstetrics has been criticized for their risks, and counter-movements have formed that seek natural, intervention-free births in order to re-naturalize births and increase the autonomy of birth mothers [10–12]. The risks associated with increased obstetrical intervention and the medicalization of childbirth need more attention. An important driver for the “counter-movements” is a growing awareness among both consumers and maternity care professionals that the increasing use of interventions during childbirth does not always improve safety. In contrast, in some cases, there is rather increased risk of adverse outcomes for the pregnant person woman and the child. Meanwhile, interventions are evaluated which seek for reducing unnecessary cesarian sections [13].

The different perspectives on births, shaped by a more natural or more medical understanding, are not only evident in professional discussions [10, 11]. Public discourse is also shaped between the contracting perspectives and conceptions of childbirth [14, 15]. Young women have to cope with this amount of information and develop individual attitudes toward childbirth. Various guidebooks and social media feature a variety of birth accounts that depict romanticized births as well as extreme birth experiences [16, 17]. Some television formats portray births as intervention-rich, dangerous, and even life-threatening [18]. Media portrayals have been hypothesized as one of the reasons for childbirth fears in pregnant women [18–20].

A German longitudinal study showed that women were more likely to receive interventions during childbirth if they had previously been convinced that childbirth was a medical process that required medical care to be managed (*medical mindset*). In contrast, pregnant women with a *natural mindset*, who assumed that birth could be managed rather independently from medical care, were more likely to deliver with low intervention rates. A low-intervention birth was experienced more positively and resulted in greater general, emotional, and physical well-being in the short term and lower rates of postpartum depressive and posttraumatic symptoms and better mother-infant bonding in the long term. Interventions occurred more frequently in the study not only among women with a medical mindset, but particularly affected first-time mothers. Birth-related cognitions and emotions are discussed as an explanation for the higher intervention rate [1, 4, 21]. Childbirth fear, which occurs in many pregnant women and is more prevalent in first-time mothers [20, 22], seems to be one such birth-obstructive emotion. Childbirth fears may come along with the wish to reduce the perceived risks of a natural birth and thus prefer medical interventions, such as cesarian section. Congruently, studies found associations between childbirth fear and cesarean sections, desire for interventions, and postpartum depressive symptoms [22]. Until now, the extent to which birth-related mindset, knowledge, and childbirth fear are related in non-pregnant women is unclear.

### Birth-related knowledge

It can be expected that experiences and information from various sources play a supporting role for the perception of birth as a natural process or in need for medical interventions [21, 23]. Knowledge could protect against childbirth fears, as it might reduce feelings of uncertainty [24–27]. It has been found that nonpregnant women without own childbirth experience use information and experiences from others [e.g. 6]. However, it is unknown what information and knowledge they have. There are only few studies that have objectively measured birth-related knowledge, but they cannot be transferred to the German population [25, 26, 28, 29]. To address this research gap, this present study developed a German knowledge test assessing young women’s knowledge of childbirth, and test for its relation to childbirth fear and mindset.

### Research goal

This study investigates, for the first time, the distribution and relationships of birth-related mindset, knowledge, and childbirth fear in young, non-pregnant women with no birth experience who will be potential first-time mothers.

Research questions are:

1. Are there differences in women with natural mindset and low fear, medical mindset and low fear, natural mindset and higher fear, and medical mindset and higher fear?
2. Is higher birth-related knowledge associated with lower fear, and a natural mindset, or is knowledge independent from fear and mindset?

First-time mothers are particularly vulnerable to fear [22] and interventions [1, 4, 21] during childbirth and its consequences. Since also non-pregnant women can be affected from childbirth fears, it is necessary to explore the meaning of potentially related aspects, i. e. birth-related mindset and knowledge especially in this group. Results from this study will give background facts which can be useful for physicians’ and midwifes’ counselling concepts for young women before pregnancy and during the perinatal period, and for reducing childbirth fears in pre-pregnant women.

## Method

The cross-sectional study was done by means of an online questionnaire. The study was approved by the Ethics Committee of the Faculty of Life Sciences of the Technische Universität Braunschweig in Germany (MA-2020-15) and is in concordance with the Declaration of Helsinki. Participants had to be female, between 18 and 40 years of age, not currently pregnant, and not yet having given birth of a viable child. A miscarriage in obstetric history of participants was no exclusion criterion. The study was conducted in autumn 2020. Young female participants were recruited online via platforms, diverse social media channels, personal contacts and flyer displays in gynecologists’ practices and piercing studios.

### Procedure and instruments

The participants first answered demographic and birth-related questions in the online questionnaire. Then they filled in the childbirth fear questionnaire (Childbirth Fear Prior to Pregnancy Questionnaire CFPP [30]), knowledge questionnaire, and the medical versus natural mindset questions (Mindset and Birth Questionnaire MBQ [33]):

### Childbirth fear

The German version of the Childbirth Fear – Prior to Pregnancy Questionnaire was used to assess birth-related fears (CFPP) [30]. The self-rating CFPP consists of 10 items, each of which is answered on a six-point Likert scale from 1 = strongly disagree to 6 = strongly agree. The questionnaire can be used with both women and men who want to have children. This study also examined women without a desire to have biological children, who were presented with the items of the CFPP in the subjunctive according to the content fit (E. g. “I am worried that labor pain will be too intense.” – “I am worried that labor pain would be too intense.”). Internal consistency was reported by the original authors to be α = .87 [30] and was also high in this present study at α = .88.

In order to compare women with rather higher or lower childbirth fear, a cut off was set according to content validity aspects: Women with a mean score > 4 were grouped as higher childbirth fear, women with a score 1–4 were grouped as lower childbirth fear. Overall mean in our investigated sample was M = 3.45 (SD = 0.95, range 1.50–5.80).

### Development of the birth-related knowledge test

To assess declarative birth-related knowledge, an objective measure should be used which has high objectivity of implementation, evaluation, and interpretation. A multiple-choice test is suitable for this purpose [31]. Since no instrument was available in German or related to the German health care system, research groups outside of Germany that have used such a measure were contacted and asked for information on its development [25, 26]. However, this information was not available, so a new knowledge test was developed in this study to capture birth-related knowledge.

The target population of this knowledge test was women between 18 and 40 years of age who were not pregnant and had not yet given birth. The knowledge test was designed to assess general knowledge about births in Germany. The aim was, on the one hand, to investigate the general knowledge of young women and, on the other hand, to differentiate between women who have a high or low level of knowledge about births. No specific level of education was assumed, but a good knowledge of German was required.

The questionnaire was based on information for expectant parents from practicing gynecologists and from the Federal Center for Health Education. Furthermore, the recommendations for intrapartum care of the WHO and educational literature for specialists in obstetrics were consulted. Official guidelines for knowledge transfer in obstetrics are not known. The material was reviewed, organized by content, and items were created from repeated themes. In forming distractors, common media misconceptions and myths were addressed, such as the assumption that after a cesarean section, all subsequent infants must also be born by cesarean section or that interventions can improve pelvic floor strength [31].

According to Bühner [32], the questions were designed as multiple-choice tasks to achieve good economy but at the same time a low guess probability. The multiple-choice tasks consisted of four content response options, of which between zero and three options were correct. In each task, it was possible to select that none of the options listed was correct. Questions about the location, duration, and pain relief of childbirth were asked in an open-ended format, because distractors did not appear to be useful for content. The question on cesarean section rate was asked in single-answer format. The first draft of the questionnaire was sent to 11 university hospital departments of obstetrics, gynecologists, and midwives with a request for comments on comprehensibility, accuracy of content, completeness, and risk of anxiety induction. Four experts returned comments. Based on these suggestions, individual items were reformulated, deleted, or newly included. Furthermore, the knowledge test was tested by five persons with regard to comprehensibility.

### Birth-related knowledge

The final version of the newly developed knowledge test consisted of 11 single- and multiple-choice items and three open-ended questions. The total score was a maximum of 45 points and was formed from the sum of correctly selected and correctly non-selected items. This method was chosen in order to quantitatively represent partial knowledge as well. A correction for correctly guessed answers was not applied, since the test did not contain a pass mark, but was intended to represent a distribution of knowledge.

The questions relate to the general process of childbirth, interventions and behavioral recommendations during childbirth. Items are different in content and there is variance in answer correctness. Accordingly, item difficulty values varied between 21 and 100 (Table 1).

**Table 1:** Questionnaire for birth-related knowledge. Correct answers are in bold letters

1	What types of facilities or places are there in Germany where women can give birth? **Hospital (outpatient/inpatient), birth center, home birth**
2	How long does a birth take on average for women giving birth for the first time? A birth in a first-time woman takes about **8–17.5 h**.
3	What are signs of the beginning of the birth? **a) gushing of amniotic fluid** **b) drop by drop of amniotic fluid** c) body temperature above 38 °C **d) discharge of mucus from the vagina**
4	During birth, the extent to which the cervix is open is checked regularly. How is this done? a) The midwife inserts a speculum (metal examination instrument) with a measuring tip into the vagina, the cervical dilation can be read. b) The midwife calculates the cervical dilation using the circumference and length of the abdomen. **c) The midwife inserts two fingers into the vagina and feels the cervical dilation.** d) The midwife performs a visual diagnosis during the vaginal examination and estimates the cervical dilation.
5	During birth, a cardiotocography (CTG) is usually done to measure labor strength and fetal heart sounds. How is this done? a) During CTG, fine measuring needles are inserted into the outer abdominal wall. **b) During CTG, a belt with ultrasound plates is placed around the abdomen.** c) During CTG, electrodes are sticked to the abdomen. d) During CTG, measuring sensors are inserted into the vagina.
6	An episiotomy refers to the surgical incision of perineal tissue to widen the birth canal. Which statement(s) is/are true? **a) An episiotomy is intended to prevent uncontrolled tearing of the tissue.** b) An episiotomy is usually necessary in first-time mothers. **c) An episiotomy is only performed when medically necessary.** d) By making the incision and then stitching the pelvic floor tightly together, the pelvic floor strength is improved.
7	What happens after the birth of the child? **a) The birth of the child is followed by the afterbirth, during which the placenta is expelled.** b) After the birth of the child, the umbilical cord must be cut immediately. c) After the birth of the child, the cervix is sutured shut again. **d) After the birth of the child, a healthy newborn may immediately go to the mother.**
8	What percentage of children are born by cesarean section in Germany? **30%**
9	What are the reasons for performing a cesarean section? a) A cesarean section is performed when clear amniotic fluid is discharged from the vagina. **b) A cesarean section is performed when the baby’s head does not fit through the mother’s pelvis.** **c) A cesarean section is performed in case of premature detachment of the placenta.** **d) A cesarean section is performed when the baby is transverse.**
10	What should be considered after a cesarean section? a) After a cesarean section, the woman must also deliver all future children by cesarean section. **b) After a cesarean section, just as with a vaginal birth, so-called regression courses are necessary.** c) After a cesarean section, the newborn is often healthier than after a vaginal birth. **d) After a cesarean section, the woman must stay in the hospital for about 5 days.**
11	What remedies are used to relieve pain during vaginal birth? **Various means from the fields of alternative methods and medicinal procedures.**
12	What are the recommendations for the birthing position? a) Women should not give birth in an upright position (e. g., standing or squatting) because the risk of falling increases the risk of head injury to the newborn. b) Whenever possible, the woman should give birth lying on her back, as this is the most efficient birthing position. **c) The birthing position can be chosen by the woman herself, although changes of position and movements are helpful for the birth process.** d) Births are performed in the bathtub by default, as this relaxes the woman’s muscles.
13	What are the recommendations for eating and drinking with regard to childbirth? a) Even small meals have a negative effect on the birth process, so women should not eat any food during birth. b) At the latest two days before the expected date of delivery, the woman should begin to increase her food intake in order to build up sufficient energy reserves for the birth. c) To prevent circulatory problems, women giving birth should at least double the amount they usually drink. d) During birth, only sugar-free foods should be consumed to prevent hyperglycemia of the child. **None of the above options is correct.**
14	What are the recommendations for going to the toilet during childbirth? a) At the beginning of the birth, an enema is given and a catheter is placed so that the bowel and bladder are emptied. **b) Often the woman can take advantage of a voluntary enema.** c) The woman must wait until after the birth to go to the bathroom. **d) Usually, due to hormonal release, there is an urge to defecate at the beginning of childbirth when the woman is still able go to the toilet on her own.**

### Birth-related mindset

The Mindset and Birth Questionnaire (MBQ) [33] was used to assess birth-related mindset. The MBQ is a self-report instrument with originally 18 items which include trust in midwives, negative view of drug support, low shame and disgust sensitivity, and positive view of vaginal birth. Items are rated on a six-point scale ranging from 1 = strongly disagree to 6 = strongly agree. The total score is calculated as the mean of all items (after inversion, if necessary). High values indicate a more natural mindset, while low values describe a more medical mindset. The internal consistency in our present study was Cronbach’s α = .73, which was acceptable for a scale which aims to describe a rather homogenous concept, i. e. a natural mindset on birth [34]. In order to compare groups of women with rather natural and rather medical mindset, a cut off was set near the present sample’s mean score (M = 4.01, SD = 0.50, normal distribution). Due to a large amount of missing in three items, we calculated the mean score over 13 items.^1^ Women with an overall mean score > 4 were grouped as having a “rather natural mindset”, women with a mean score ≤ 4 were grouped as having a “rather medical mindset”.

### Data analysis

Data have been analyzed with SPSS version 26. For investigating direction and strength of relations between characteristics, Spearman correlation analysis have been calculated (Table 3). Differences between the groups with high or low childbirth fear and birth-related knowledge have been investigated by means of analysis of variance for degrees or characteristics (ANOVA). Comparison of frequencies have been done for categorial characteristics (Chi^2^).

## Results

### Distribution of childbirth fear and birth-related mindsets

Half of the 316 women (51.6%) had a natural birth-related mindset, and 27% of women reported a relevant level of childbirth fear (Table 2). Considering these two relevant aspects together, most women (44%) had a natural mindset and low childbirth fear, 29% had a medical mindset and low childbirth fear, 8% natural mindset and higher childbirth fear, and 19% medical mindset and higher childbirth fear (Table 2).

**Table 2:** Childbirth fear, birth-related natural mindset and knowledge of young women before their first birth (*N* = 316)

Characteristics	Natural Birth Mindset, Low Fear Nf (***n*** = 138)	Medical Birth Mindset, Low Fear Mf (***n*** = 92)	Natural Birth Mindset, Higher Fear NF (***n*** = 25)	Medical Birth Mindset, Higher Fear MF (***n*** = 61)	Significance of difference between the groups ***p*** overall (ANOVA or Chi^**2**^ Test)
**Age**	23.43 (3.29)	23.67 (3.37)	22.36 (2.98)	24.11 (4.04)	.183
**Educational Level** % High School (12 classes) or University	97.8%	96.7%	96.0%	95.1%	.581
**Relationship or Married** %	62.3%	61.9%	80.0%	70.5%	.446
**Own birth modus**					.404
Normal birth	82.6%	72.8%	72.0%	80.3%	
Cesarian Section	15.9%	21.7%	20.0%	14.8%	
Suction Cup/Pliers	0.7%	4.3%	4.0%	4.9%	
Don’t know	0.7%	1.1%	4.0%	0.0%	
**Watched another birth** %	13.0%	3.0%	4.0%	3.3%	.016
**Earlier Pregnancies** %	3.6%	1.1%	0.0%	1.6%	.482
**Childbirth Fear** (CFPP mean, scale 1–6)	2.86 (0.67)	3.21 (0.65)	4.50 (0.36)	4.71 (0.42)	.000
**Birth-related Natural Mindset** (MBQ mean, scale 1–6)	4.39 (0.32)	3.62 (0.32)	4.30 (0.24)	3.34 (0.48)	.000
**Birth-Related Knowledge** Sum Score (0–45 Points) Empirical range in whole sample: 22–44	34.10 (3.89)	33.20 (3.41)	34.08 (4.13)	32.93 (3.66)	.124
**Sources of Knowledge**					
Visual media (TV)	82.6%	71.7%	88.0%	78.7%	.154
Text media (books, articles)	70.4%	64.1%	60.0%	60.7%	.486
Friends’ reports	41.4%	29.7%	9.0%	20.0%	.851
Family’s reports	85.5%	89.1%	80.0%	77.0%	.205
Sexual education in school	60.9%	55.4%	56.0%	65.6%	.617
Professional education	5.8%	2.2%	4.0%	4.9%	.624

All of the women answered at least 50% of the knowledge questions correctly, 48.8% achieved at least 34 points (i. e. 75% correct answers), but only 2.7% answered 90% correctly (41 or more points in the knowledge questionnaire).

Gaps in knowledge appeared especially concerning signs of beginning of birth (22.9% correct answers only), and non-medical approaches to pain relief: 20% did not know any pain reduction method and only 11.7% knew non-medical approaches. More than 50% of women did not know the duration of birth (8–17 h), and that 30% of births happen by cesarian section. Well known were possible places of birth (correct answers by 87.5% of women), indication for perineal cut (91.8%), what happens after birth (73.3%), reasons for cesarian section (83.7%), and what happens after (83.9%), how to manage eating and drinking before birth (87.2%) and going to the toilet (76.0%).

There were no significant differences in birth-related knowledge, sources of knowledge, and education level between the four groups (Table 2). Similarly, there were no systematic differences in distribution of the women’s own birth modus, relationship status, or earlier (lost) pregnancies. But, from women with natural mindset and low childbirth fear, a higher percentage (13%) has already watched a birth, as compared to the other groups.

### Relationships of birth-related knowledge, childbirth fear and mindset

Additional correlative analysis showed that a natural mindset was moderately associated with lower childbirth fear (*r* = −.425**, Table 3), whereas knowledge was independent from childbirth fear (*r* = −.051). Higher knowledge was low associated with natural mindset (*r =* .165**). Mindset and childbirth fear were independent from age and education degree.

**Table 3:** Spearman correlations of childbirth fear, natural mindset, and knowledge in young women (*N* = 316)

	1	2	3	4
**1 Age**				
**2 Education degree**	.533**			
**3 Childbirth Fear**	.026	−.064		
**4 Birth-related Natural Mindset**	−.081	.013	−.425**	
**5 Birth-related Knowledge**	.201**	.123*	−.051	.165**

*p<.05, **p<.01

## Discussion

### Childbirth fear and mindset

The first relevant result is that there were hardly differences between the four groups: women with high childbirth fear and natural mindset, or medical mindset, and women with low childbirth fear and natural mindset or medical mindset. The only difference was a higher rate of having observed a birth in the group of natural mindset and low childbirth fear. Our cross-sectional study does not allow causal interpretations, but hypotheses can be drawn from these findings: It may be that women with low fear are more prone to expose themselves to watch a birth, or women who watch a birth may get a reframing of potential worries or vague ideas they had on birth before. While watching a birth, women are confronted with real life examples of what a birth can be like. Psychologically, aspects of model learning and holistic learning may apply hereby, be it a natural birth or medically supported birth. Model learning and holistic observation may both change the perception and knowledge on birth, from abstract or theoretical facts to realistic impression of what a birth may be like.

### Relationships between childbirth fear, knowledge and mindset

#### Childbirth fear and mindset

Lower childbirth fear came along with rather natural mindset.

Even if the relationship is not very strong, the tendency fits to what has been found and hypothesized in the literature before [1, 4, 21].

#### Childbirth fear and knowledge

Childbirth fear occurred independent from the level of birth-related knowledge. This result is similar to an earlier correlative study which found that birth-related knowledge and childbirth fear were independent from each other [40]. However, there are also contrary findings which report a negative association [25–27, 41]. The discrepancy of our present findings and findings indicating an association of knowledge and anxiety may be due to several aspects. First, cross-cultural differences must be considered, as both birth care and birth culture vary greatly between countries [42]. The lack of standardized measures of knowledge means that the different levels of knowledge cannot be compared between samples and cannot be conclusively classified. In the randomized controlled trial [25] not only pure factual knowledge was taught, but also discussions and various exercises on relaxation and breathing techniques were conducted. Thus, in addition to increased knowledge, other factors that may have led to a reduction in childbirth fear, such as a sense of self-efficacy, social support, and the development of action skills, must be considered. Furthermore, studies on childbirth fear were able to show contrasting behaviors related to knowledge, indicating opposing associations; some women with childbirth fear specifically sought information, while others avoided it or worried about their knowledge [22].

The availability of individual knowledge elements in everyday life may also contribute to the independency of childbirth fear and knowledge. The birth-related mindset may lead to heuristic use of knowledge. Even if comprehensive knowledge can be mapped in the knowledge test, this does not mean that it is used for own birth-related decision-making.

#### Birth-related mindset and knowledge

There was hardly an association between birth-related mindset and birth-related knowledge. It is assumed that the birth-related mindset is based on different information [21]. A higher birth-related knowledge could however be supportive for a rather natural mindset. Therefore, a natural view of births could be strengthened by giving psychoeducation and knowledge on normal childbirth [36, 43, 44].

### Limitations

The study is cross-sectional, which means that no causal conclusions can be made from the data.

In the present study, it was not assessed whether the women suffered from any mental disorder in general or even an anxiety disorder in particular. A mental disorder or particularly anxiety disorder might however impact on birth-related fear, in the sense that some of the women with high general anxiety or anxiety disorder might perceive childbirth anxiety as well. Similar observations have been made with other specific anxieties, such as specific work-anxiety accompanying general mental disorders in some patients [45]. Also, half of women with specific phobias, including birth-related phobias were found to have other general (not birth-related) mental disorder [5].

In this study we used self-ratings to assess the degree of childbirth fear and natural and medical mind set. Self-reporting scores may not reflect actual emotions, nor report actual needs of a non-pregnant woman. For clinical diagnostic or decision making the full range of biopsychosocial diagnostics by health care professionals and physicians is necessary.

The knowledge test was developed for this study, in order to be an indicator of what the women know about birth (instead of what they think they know, in the sense of “I am convinced that I know a lot about birth”). Although the contents of the test and the correctness of answers have been prepared very carefully with several expert consultants, it may be that in different regions or different medical traditions other issues seem to be more important and other contents would have been asked or other answers offered.

Furthermore, our choice of instruments may influence the results in terms of childbirth fear rates. Alternative scales or also clinical interviews might be used in further research. An alternative self-rating questionnaire could be the Wijma Delivery Expectancy-Experience Questionnaire (W-DEQ) [46] which asks women about their expectations before the delivery (version A) and experience after delivery (version B).

### Perspectives for practice and research

#### Prevalence and diagnostics

Most of the investigated non-pregnant women (43.6%) of our study had a natural mindset and at the same time low childbirth fear. The rate of 27% of our study participants with higher childbirth fear appears similar to the rate of 25.9% childbirth fear in non-pregnant female students which was found by Antic [6]. Differences in prevalence estimations may be due to the different samples (pregnant, non-pregnant women), methodologies (structured interviews, self-rating questionnaires, different cut-off scores), and variance in cultural and sample characteristics [35, 36].

The rate of childbirth fear in our sample is similar to rates of general mental health problems in the general population which is constantly about 30%, covering different unspecific mental health problems [39]. It may be that non-pregnant women with mental disorders who react with anxiety in several life situations, are also more likely to experience birth-related fear. Studies in pregnant women reported childbirth fear as associated with stress, depression, anxiety, or history of mental disorder [36, 37]. Also, fathers may be affected (13%) [38].

However, the pattern of relation of knowledge, mindset and childbirth fear may be very different in each single case. In clinical practice of specific cases, a thorough clinical investigation of the women is necessary. Especially in cases of phobic anxiety with clinical value and need for intervention a thorough anamnesis is mandatory. Self-rating questionnaires are useful for research and observation of symptom load in different samples or over the course of an intervention, but not for making a clinical diagnosis of childbirth fear.

#### Childbirth fear, mindset and birth-related knowledge in pregnancy counselling

As childbirth fear may occur in different pattern (i.e. with natural or medical mindset), such fears should be taken earnest and explored in non-pregnant women with desire for pregnancy. As birth-related mindset is related with knowledge, and women with natural mindset less often have childbirth fears, it may be fruitful to offer some facts about natural aspects of birth (e.g. such as the contents from the here developed knowledge test), or give the opportunity to observe educative birth videos or midwife-attended natural births in which overemphasize of medical interventions is omitted [47]. This would be in line with intervention studies which give a hint that self-efficacy-oriented interventions, such as physicians education, and childbirth workshops for women or even couples [13], may increase women’s choice for spontaneous vaginal births (instead of choosing cesarian sections). Furthermore, some knowledge gaps were found in our sample concerning signs of beginning birth, and non-medical approaches to pain relief. These could be topics of interest in psychoeducation for non-pregnant women.

#### Research

Our research – beside other [6] - has shown that also non-pregnant women can be affected from childbirth fears, and these are partly related with the birth-related mindset. Thus, further research should investigate whether and which aspects from existing non-medical educative interventions are useful and adaptable for non-pregnant women with desire for having children.

## Conclusion

Most of the investigated women had a natural birth-related mindset and low childbirth fear.

Nonetheless, one in four women showed higher levels of childbirth fear, most of whom had a rather medical mindset.

In counseling with patients, practitioners should be aware of childbirth fear and try to develop an awareness for birth as a normally natural event, e.g. by using self-efficacy-oriented educational and informative non-medical interventions [13, 43, 44, 47].

## Data Availability

Data are available from the authors upon request.

## Footnotes

1 The affected items are from the scales Trust in midwives (Item 16 and Item 18) and Negative view of drug support (Item 17). These data failures are missing completely at random and are not related to content factors. Because of the high number of data dropouts, we did not impute the scores. Because each scale continued to have at least three items, a calculation of internal consistencies was performed for the complete and abbreviated forms within the subsample that had the MBQ presented in its entirety (*n* = 101). The internal consistencies differed only marginally, with Cronbach’s alpha of α = .82 for the full version and α = .81 for the abbreviated version, so it can be assumed that the abbreviated version also depicts the construct with equal reliability. The difference between the scores formed by the complete and by the shortened version was on average M = 0.04 (SD = 0.14), so that the scores formed by the complete version were on average insignificantly higher. In addition, an examination of the structure of the abbreviated form was performed using principal component analysis and varimax rotation in the total sample. The same structure identified by Hoffmann (2020) [21] was found, and all items could be combined into the intended scales. Because both the reliability analysis and principal component analysis results were satisfactory, the shortened version of the MBQ within the total sample was used for data analysis.
